# Quantitative Genetics of Body Size and Timing of Maturation in Two Nine-Spined Stickleback (*Pungitius pungitius*) Populations

**DOI:** 10.1371/journal.pone.0028859

**Published:** 2011-12-14

**Authors:** Yukinori Shimada, Takahito Shikano, Anna Kuparinen, Abigél Gonda, Tuomas Leinonen, Juha Merilä

**Affiliations:** Ecological Genetics Research Unit, Department of Biosciences, University of Helsinki, Helsinki, Finland; Auburn University, United States of America

## Abstract

Due to its influence on body size, timing of maturation is an important life-history trait in ectotherms with indeterminate growth. Comparison of patterns of growth and maturation within and between two populations (giant *vs.* normal sized) of nine-spined sticklebacks (*Pungitius pungitius*) in a breeding experiment revealed that the difference in mean adult body size between the populations is caused by differences in timing of maturation, and not by differential growth rates. The fish in small-sized population matured earlier than those from large-sized population, and maturation was accompanied by a reduction in growth rate in the small-sized population. Males matured earlier and at smaller size than females, and the fish that were immature at the end of the experiment were larger than those that had already matured. Throughout the experimental period, body size in both populations was heritable (*h^2^* = 0.10–0.64), as was the timing of maturation in the small-sized population (*h^2^* = 0.13–0.16). There was a significant positive genetic correlation between body size and timing of maturation at 140 DAH, but not earlier (at 80 or 110 DAH). Comparison of observed body size divergence between the populations revealed that *Q*_ST_ exceeded *F*_ST_ at older ages, indicating adaptive basis for the observed divergence. Hence, the results suggest that the body size differences within and between populations reflect heritable genetic differences in the timing of maturation, and that the observed body size divergence is adaptive.

## Introduction

In many organisms, and in particular in ectotherms with indeterminate growth, age and size at maturation are among the most important life-history traits affecting fitness [1]–[3]. There is a trade-off between these two traits: while early maturation decreases the probability of dying before reproduction, it also entails smaller size and thereby lowered fecundity especially in females (e.g. [4]–[6]). In contrast, delayed maturation increases the risk of death before reproduction, while it also increases fecundity through increased size at maturation (e.g. [4]–[6]). Therefore, in environments with high mortality rates, such as in populations where individuals are subject to intensive predation, early maturation at smaller size is expected to evolve as compared to populations with lower mortality risks [1], [2], [7].

Both the proximate determinants of timing of maturation and somatic growth rate are known to be influenced by environmental and genetic effects (e.g. [8]–[10]). As to the environmental effects, low temperatures are known to reduce growth and developmental rates in a wide variety of organisms ranging from bacteria and protists to plants and animals [8], [11]. Yet, slower development and delayed maturation caused by low temperatures typically result in increased final body size [8], [11]. As to the genetic effects, the genetic basis for variation in somatic growth rates in fish is well established even in the wild. Heritability estimates for growth rate range from moderate (*h^2^*≈0.2) to high (*h^2^*≈0.8) in various species of fishes (e.g. [12]–[14]). However, heritability estimates (*h^2^* = 0–0.67) for timing of maturation in fish are still quite rare ([15]–[21]; reviewed in [9]), as are estimates from other ectotherms (e.g. [22], [23]). Hence, the timing of maturation is expected to influence body size through its effect on growth rate – this is due to energy being partly allocated to reproductive processes instead of somatic growth only [24]. However, little is known about the genetics of maturation and its role in determining final body size (but see: [25]–[27]). Specifically, it is debatable whether maturation can evolve independently of growth or whether the timing of maturation is also linked to growth preceding maturation (e.g. [27]). In the latter case, evolutionary shifts in maturation would be accompanied with changes in growth trajectories. On the other hand, if maturation could evolve independent of growth, individuals with differing maturation schedules could have similar initial growth trajectories. For example, the probabilistic maturation reaction norm approach, which is often used in the analyses of fisheries-induced evolution, relies on the assumption that variation in growth is largely environmental and that genetic changes in maturation are seen after controlling for changes in growth (e.g. [27], [28]).

The nine-spined stickleback (*Pungitius pungitius*) is a small freshwater fish that typically reaches a total length of 5–6 cm [29]. However, gigantism occurs in some Fennoscandian ponds, in which adults attains body size twice as large as nine-spined sticklebacks in other populations [30]–[32]. Although heritabilities and the influence of maternal effects on growth and body size in this species have never been estimated, common garden experiments have demonstrated that gigantism indeed has a genetic basis [30], [31]. However, whether this gigantism results from a faster growth rate or from a prolonged growth period remains to be investigated (but see [31]), as does the question of whether the attainment of maturity is accompanied by reduced growth rates. Namely, if there is a trade-off between maturation and size at maturity – as suggested by earlier work in other species (e.g. [4]–[6], [33]) – one would expect to observe that initiation of maturation slows growth rates. According to this expectation, differences in timing of maturation could at least partly explain body size differences – and occurrence of gigantism – among and within nine-spined stickleback populations.

The aim of this study was to compare growth and timing of maturation in two phenotypically contrasting (*viz*. giant and normal sized) nine-spined stickleback populations under common garden conditions. In particular, we were interested in exploring if delayed timing of maturation could be a possible evolutionary driver of intraspecific gigantism in the large-sized population. Additionally, we aimed to determine whether the giant pond population – known to have lost most of its genetic variability in neutral marker genes [34] – is also lacking in additive genetic variance for phenotypic traits. To this end, we produced half-sib families in both populations and reared individuals up to an age of 140 days in a common garden experiment. To probe possible genetic trade-offs, we also estimated genetic correlation between body size and timing of maturation at different time-points in the small-sized population. Growth, timing of maturation, and genetic parameters within and between populations were compared at five intervals throughout the developmental period. To establish whether differentiation between populations was adaptive, we compared phenotypic differentiation (*Q*_ST_) with neutral expectation (*F*_ST_) as estimated from common garden data and neutral microsatellite loci, respectively [35], [36].

## Results

### Growth and timing of sexual maturation

**Figure 1:**
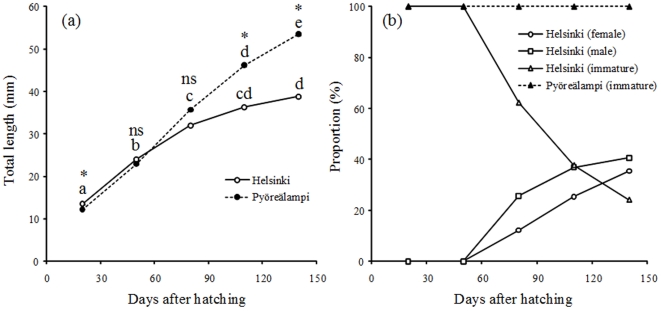
Patterns of (a) growth and (b) maturation in two nine-spined stickleback populations as a function of time since hatching. In (a) the plotted values are means (± S.E.) and asterisks (*) indicate significant (*P*<0.001) difference between means (ns = not significant). Means labelled with different letters are significantly different from each other. In (b) the plotted values are proportions of mature and immature individuals by sex in each population (in case of the immature, sex is not known).

The patterns of growth differed between the two populations: while the mean body size of Pyöreälampi individuals increased more or less linearly throughout the observation period, the growth of the Helsinki fish began to slow down at 80 days after hatching (DAH; Fig. 1a). Pyöreälampi fish were significantly smaller than Helsinki fish at 20 DAH (likelihood ratio test: *LRT* = 8.1, *P* = 0.005), whereas at 50 and 80 DAHs no significant difference could be detected (50 DAH: *LRT* = 1.9, *P* = 0.166; 80 DAH: *LRT* = 0.45, *P* = 0.504). At 110 and 140 DAHs Pyöreälampi fish were significantly larger than Helsinki fish (*LRT* = 63.2, *P*<0.001, and *LRT* = 115.7, *P*<0.001, respectively). Variance components for family at 20, 50, 80, 110 and 140 DAHs were 65.8%, 9.0%, 12.8%, 7.7% and 9.6%, respectively, whereas the respective numbers for block effects were 1.4%, 32.0%, 6.6%, 4.8%, and 4.0%, respectively. The declining growth rate in the Helsinki population at 110 DAH coincided with the onset of maturation, when 62% had matured at that stage and 76% at the end of the experiment (Fig. 1b). In contrast, no Pyöreälampi individuals matured during the experiment (Fig. 1b). Consequently, in the analyses of the probability of maturation by 80 and 110 DAHs, population had a significant and substantial effect on maturation (80 DAH: *D* = 86.2, *df* = 1, *P*<0.001; 110 DAH: *D* = 10.7, *df* = 1, *P*<0.001). In addition, the probability of maturing by 80 DAH was positively correlated with total length at 50 DAH (*D* = 15.9, *df* = 1, *P*<0.001), but negatively correlated with total length at 80 DAH (*D* = 4.7, *df* = 1, *P* = 0.031). Similarly, the probability of maturing by 110 DAH was positively correlated with length at 50 DAH (*D* = 4.8, *df* = 1, *P* = 0.028) and with length at 80 DAH (*D* = 7.0, *df* = 1, *P* = 0.008), but negatively correlated with length at 110 DAH (*D* = 34.0, *df* = 1, *P*<0.001). Density at 50 DAH had no effect on the probability of maturing by 80 DAH (*D* = 0.9, *df* = 1, *P* = 0.337), whereas density at 80 DAH correlated negatively with the maturation probability at 80 DAH (*D* = 5.3, *df* = 1, *P* = 0.020). Density at 50 DAH and 80 DAH had no effect on the probability of maturing by 110 DAH (50 DAH: *D* = 0.03, *df* = 1, *P* = 0.857; 80 DAH: *D* = 0.25, *df* = 1, *P* = 0.620), whereas density at 110 DAH correlated negatively with the maturation probability (*D* = 10.7, *df* = 1, *P*<0.001). Among the Helsinki fish, males matured earlier than females (Fig. 1b) and although more males (n = 234) than females (n = 204) matured before the end of the experiment, the sex ratio of the mature individuals did not differ from 1∶1 expectation (Chi-square test: *χ* ^2^ = 0.896, *df* = 1, *P* = 0.344). Hence, the fish that were immature at the end of the experiment (24%) were unlikely to be mostly females.

### Sex differences in size

**Figure 2:**
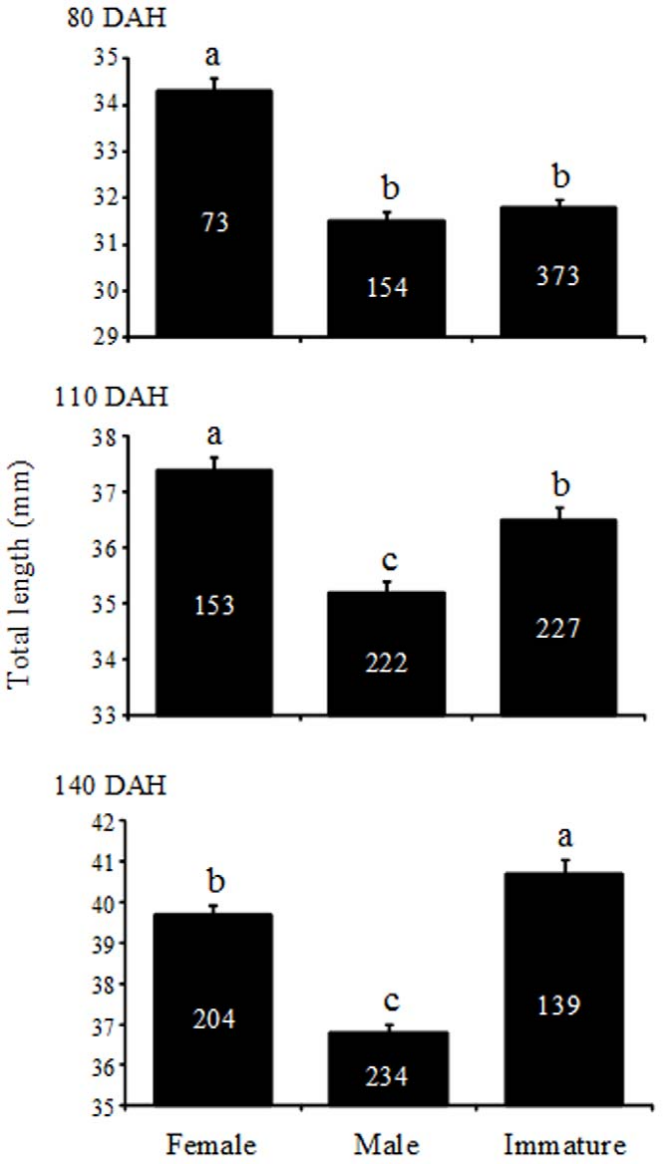
Mean body size (total length ± S.E.) of Helsinki males, females and immatures at three different ages (80, 110 and 140 days after hatching [ = DAH]). Sample sizes are shown in each bar. Means labelled with different letters are significantly different from each other.

If early maturation slowed down growth, one would expect to see that i) males – which mature earlier than females (see above) – would be smaller than females, and ii) the fish not reaching maturity by the end of the experiment would be larger than those that matured. As expected, at all different time points where comparison of body sizes between sexes were possible, females were larger than males (80 DAH: *LRT* = 51.5, *P*<0.001; 110 DAH: *LRT* = 58.6, *P*<0.001; 140 DAH: *LRT* = 89.5, *P*<0.001, see Fig. 2). Likewise, at the end of the experiment (140 DAH) immature individuals were significantly larger than males (*LRT* = 143.0, *P*<0.001) and females (*LRT* = 23.9, *P*<0.001; Fig. 2). At 80 and 110 DAHs, immature individuals were smaller than females (80 DAH: *LRT* = 40.3, *P*<0.001; 110 DAH: *LRT* = 11.3, *P*<0.001), and similar (80 DAH; *LRT* = 3.7, *P* = 0.055) or larger (110 DAH; *LRT* = 22.0, *P*<0.001) than males (Fig. 2).

### Genetics of body size and timing of maturation

**Figure 3:**
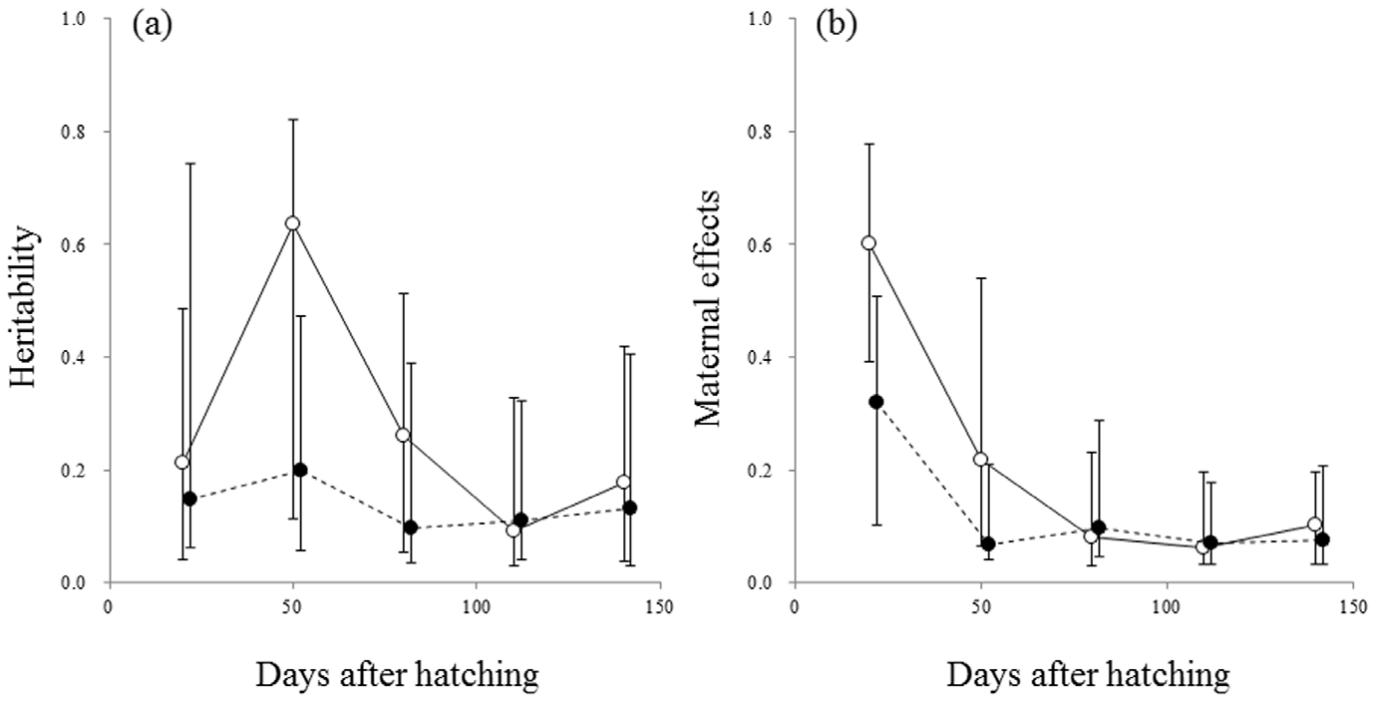
Temporal changes of heritability estimates (a) and maternal effects (b) in two nine-spined stickleback populations. Vertical bars are HPD 95% C.I.

Heritability estimates (*h^2^*) for body size in the Helsinki population were significant at all ages, and varied from 0.09 to 0.64 (Fig. 3a; Table 1). Similarly, *h^2^* estimates for body size in the Pyöreälampi population were significant at all ages and varied from 0.10 to 0.20 (Fig. 3a; Table 1). Heritability estimates for body size tended to be similar between the two populations, as revealed by the overlapping confidence intervals (Fig. 3a; Table 1). Also, the influence of maternal effects, including both maternal genetic and environmental effects, on body size were similar in the two populations (Fig. 3b); they were highest at 20 DAH in both populations, declined drastically by 50 DAH and then remained low thereafter (Fig. 3b and Table 1). Heritability estimates for the timing of maturation in the Helsinki population were significant and moderate (*h^2^* = 0.13–0.16; *m^2^* = 0.11–0.14) at 80, 110 and 140 DAHs (Table 2). Estimation of heritability was not possible for any time-point in Pyöreälampi population, where no maturation was observed (see above). As to the maternal effects, a formal comparison of the full model with its appropriate restricted model (cf. [37]) revealed no statistical evidence for maternal effects on maturation at any of the time points. However, the overall pattern of estimated maternal effects was concordant with the general pattern observed in earlier studies of fishes (e.g. [38], [39]): that is, large influence of maternal effects at younger ages, and declining thereafter (Table 1; Fig. 2b).

**Table 1:** Causal components of variance and heritability (*h* ^2^) of body size at different ages (in days) in two nine-spined stickleback populations.

Population	Age	N	*V*_A_	*V*_M_	*V*_P_	*h* ^2^	*m* ^2^
Helsinki	20	712	0.8 (0.3; 2.5)*	2.6 (1.4; 5.8)	4.8 (3.5; 7.6)	0.21 (0.04; 0.49)	0.60 (0.39; 0.78)
	50	672	5.0 (1.2; 7.9)	1.7 (0.3; 6.2)	8.7 (6.8; 12.9)	0.64 (0.11; 0.82)	0.22 (0.06; 0.54)
	80	600	2.0 (0.4; 4.5)	0.8 (0.2; 1.9)	8.0 (6.9; 9.8)	0.26 (0.05; 0.51)	0.08 (0.03; 0.23)
	110	602	0.9 (0.3; 3.4)	0.8 (0.3; 2.0)	9.5 (8.2; 11.1)	0.09 (0.03; 0.33)	0.06 (0.03; 0.20)
	140	577	2.2 (0.5; 5.9)	0.9 (0.4; 2.7)	12.3 (11.0; 15.1)	0.18 (0.04; 0.42)	0.10 (0.03; 0.20)
Pyöreälampi	20	1563	0.3 (0.1; 1.8)	0.6 (0.2; 1.3)	2.1 (1.8; 2.9)	0.15 (0.06; 0.74)	0.32 (0.10; 0.51)
	50	1049	0.9 (0.2; 2.4)	0.3 (0.2; 1.0)	4.4 (3.9; 5.4)	0.20 (0.06; 0.47)	0.07 (0.04; 0.21)
	80	275	0.9 (0.3; 4.0)	0.9 (0.4; 3.0)	8.8 (7.4; 11.6)	0.10 (0.04; 0.39)	0.10 (0.05; 0.29)
	110	253	1.5 (0.5; 5.2)	1.0 (0.5; 2.9)	15.7 (12.3; 18.0)	0.11 (0.04; 0.32)	0.07 (0.03; 0.18)
	140	244	1.6 (0.4; 6.9)	1.1 (0.5; 3.4)	14.7 (11.8; 18.1)	0.13 (0.03; 0.40)	0.08 (0.03; 0.21)

*mode (HPD 95% C.I.: low; up), *V*_A_ = additive genetic variance, *V*_M_ = maternal effect variance, *V*_P_ = phenotypic variance, *m* ^2^ = proportion variance explained by maternal effects.

**Table 2:** Causal components of variance and heritability (h2) for timing of maturation (matured: 0, immature: 1) in Helsinki population.

Age	N	*V*_A_	*V*_M_	*V*_P_	*h* ^2^	*m* ^2^
80	600	0.009 (0.004; 0.022)*	0.008 (0.003; 0.014)	0.049 (0.044; 0.061)	0.16 (0.08; 0.38)	0.14 (0.07; 0.24)
110	602	0.009 (0.004; 0.024)	0.007 (0.004; 0.016)	0.072 (0.060; 0.082)	0.13 (0.07; 0.31)	0.13 (0.06; 0.21)
140	577	0.010 (0.004; 0.029)	0.008 (0.003; 0.018)	0.066 (0.057; 0.081)	0.15 (0.06; 0.39)	0.11 (0.07; 0.25)

*mode (HPD 95% C.I.: low; up), *V*_A_ = additive genetic variance, *V*_M_ = maternal effect variance, *V*_P_ = phenotypic variance, *m* ^2^ = proportion variance explained by maternal effects.

There was a significant positive genetic correlation between body size and timing of maturation at 140 DAH in Helsinki population (*r*_g_ = 0.874, 95% HPDI = 0.725–0.954). In other words, the later the individuals matured, the larger they were genetically. Genetic correlation estimates at other time-points were non-significant (80 DAH: *r*_g_ = 0.240, 95% HPDI = −0.457–0.610; 110 DAH: 0.013, 95% HPDI = −0.607–0.719).

### Quantitative genetic differentiation of body size

**Figure 4:**
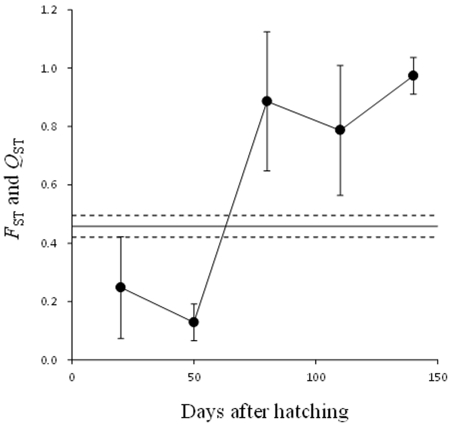
Comparison of the degree of quantitative trait divergence (*Q*_ST_) in body size and divergence in neutral microsatellite loci (*F*_ST_) at different ages. Filled circles and vertical bars are posterior means of *Q*_ST_ values and their standard deviations, respectively. Horizontal line and dotted lines represent the mean *F*_ST_ estimate and its standard error (0.458±0.019), respectively.

The quantitative genetic differentiation of body size at different ages, as measured by *Q*_ST_, was estimated to range from 0.128±0.063 (mean ± S.D.) at 50 DAH to 0.973±0.063 at 140 DAH (Fig. 4). The drastic increase in *Q*_ST_ estimate at 80 DAH coincides with the maturation of the fish from the small-sized population: at 20 and 50 DAHs the *Q*_ST_s were clearly lower than the neutral genetic expectation set by *F*_ST_ (0.458±0.019: mean ± S.E.), whereas at 80, 110 and 140 DAHs the *Q*_ST_s were higher than the *F*_ST_ (Fig. 4).

## Discussion

Our common garden experiment demonstrated that nine-spined sticklebacks from the pond population (Pyöreälampi) reach a larger body size than those from the coastal population (Helsinki) – a finding that parallels results reported in earlier comparisons of both wild and common garden reared nine-spined sticklebacks [30]–[32]. However, it was revealed that fish from the pond population were not larger throughout the entire experimental period, but were in fact initially smaller than the fish from the coastal population. This size rank reversed at about 80 DAH, at which point the fish from the coastal population started to mature, while fish from the pond population remained immature. Even though larger body size increased the probability of maturation, a substantial population component in the onset of maturation remained even after controlling for the effect arising from differences in growth. Therefore, even though early maturation was to some extent linked to faster initial growth, different maturation schedules in the two populations could not be solely explained by differences in growth. Rather, a substantial growth-independent component was also present. Generally, it appears that population differentiation in maturation schedule (rather than differentiation in their growth strategies) explains the population differentiation into giant *versus* normal body size: the later the fish mature, the larger they will grow. This pattern, revealed by the inter-population comparison, was paralleled by an intra-population comparison of the Helsinki fish. Here, we also observed that immature individuals were larger than mature individuals when fish at later DAHs were compared. Moreover, we found a significant positive genetic correlation between body size and timing of maturation at 140 DAH, but not at earlier stages. Hence, immature fish that delay their timing of maturation are likely to become larger than those fish which have already matured. These results align with the view that delayed maturation generally increases body size in ectotherms (e.g. [4]–[6], [33], [40]–[42]).

We found that both body size and timing of maturation were heritable in nine-spined sticklebacks. This is not surprising as body size is also shown to be heritable in a number of three-spined stickleback populations (e.g. [43]–[46]), as well as in populations of many other species of fish (e.g. [9], [15], [17]–[20], [47]–[49]). However, given that the giant-sized population is known to have lost most of its genetic variability in neutral microsatellite markers (Helsinki: expected heterozygosity, *H*_E_ = 0.590 and Pyöreälampi: *H*_E_ = 0.004; [34]) we expected to see a reduction of additive genetic variance in Pyöreälampi population. Yet, no evidence of this was found, as heritabilities for body size were similar in both populations throughout the experimental period. This disconcordance between marker and quantitative trait variability has been observed also in earlier studies (e.g. [50]). Such findings are not entirely unexpected given the large variance that is seen in the level of reduction in additive genetic variance of different traits among replicate lines subject to similar levels of inbreeding [51]. Furthermore, traits coded by many genes – such as body size – may be more susceptible to pleiotropy and epistatic effects than traits coded by fewer genes [52]. Because population bottlenecks can convert pleiotropic and epistatic variance to additive variance, heritabilities are not necessarily reduced during population bottlenecks, but can even be increased [53], [54].

We found that timing of maturation was heritable in the Helsinki population of nine-spined sticklebacks (the only population available for this analysis, since the Pyöreälampi population did not reach maturation). This is an interesting finding, since heritability estimates for timing of maturation in fish are still scarce (but see: [15]–[21]). The finding is of additional interest, because the timing of maturation is a trait under natural (e.g. [16], [17], [19]) and fisheries-induced selection (e.g. [9], [55]). For example, fisheries-induced mortality is expected to favour early maturation and lead to evolution of early maturing fish (e.g. [9], [56], [57]). The degree of genetic *vs.* environmental factors in driving these observed shifts in timing of maturation remains a contested issue, particularly in the case of exploited fish populations. This is largely due to the difficulty of assessing genetic basis for the observed shifts (e.g. [28], [55], [58]). Our results showed that the two study populations have diverged genetically in the timing of maturation. This result suggests that genetic shifts in maturation schedules are possible without substantial shifts in growth trajectories preceding maturation, and that timing of maturation in nine-spined sticklebacks could be – at least in principle – evolvable in the face of selection. However, in order for this to happen, maturation has to be heritable at the time when the selection is taking place. This scenario also depends on environmental conditions: environmental effects can change heritabilities (e.g. [59]) and mask or reverse genetic trends (e.g. [60]).

Given that estimates for heritability of timing of maturation are scarce, it is not surprising that estimates of genetic correlation between timing of maturation and body size are even more scare (but see: [61]). We found a positive genetic correlation between body size and timing of maturation suggesting that delayed timing of maturation is genetically linked to increased body size. This finding parallels that of Páez *et al.* [61] who reported a positive genetic correlation between body size and timing of maturation in the Atlantic salmon (*Salmo salar*) males. These positive genetic correlations may not be surprising, but they highlight the fact that size and age at maturation may be fairly tightly genetically coupled, and selection acting one of the traits can be expected to lead to correlated responses in another.

The observed degree of differentiation in body size between pond and coastal populations exceeded that expected from genetic drift alone, and hence, is most likely due to directional selection that favors different optima for body size in each of the two populations. This aligns with an earlier inference from this same system (e.g. [31], [32]), and is particularly noteworthy given high ‘baseline’ level of neutral genetic differentiation among these populations (*F*_ST_ = 0.458±0.019; [62]). More importantly, the estimated degree of differentiation increased drastically with increasing age: at early ages (until 50 DAH), *Q*_ST_ estimates tended to be lower than *F*_ST_ estimates suggesting stabilizing selection for body size (cf. [35], [36]). However, from 80 DAH onwards, directional selection was suggested to prevail, as revealed by *Q*_ST_ estimates which were higher than *F*_ST_ estimates. Although ontogenetic patterns in *Q*_ST_ estimates have seldom been investigated (but see [63]), it seems likely that similar patterns can be expected to be found whenever growth trajectories of populations are ontogenetically divergent.

A genetic difference in the mean body size among populations can be reached in different ways, either by differences in growth rate or length of growth period [26], [27]. In other words, a large asymptotic size can be reached either by growing fast per time unit (i.e. large *k*), or by extending the growth period. In our experiment the pond fish reached larger body sizes by extending their growth period. This finding is in slight discordance with earlier results of similar experiments from this population, where the pond fish had both higher growth rates and prolonged growth period [31]. However, this difference could be attributed to differences in experimental setup between studies: our fish were reared in groups, whereas those of the earlier study were reared individually. It has been shown that group-reared nine-spined sticklebacks from pond populations grow ca. 10% slower than their individually reared counterparts [64]. However, this does not apply to marine nine-spined sticklebacks, which grow at the same rate whether reared in groups or individually [64]. This habitat-specific effect of individual *vs.* group rearing is apparently due to population differences in sensitivity to intraspecific interactions, where pond fish are more aggressive [65] and thus more constrained by interference from conspecifics [64]. Nevertheless, even if the group-rearing environment of our experiment potentially depressed growth rates, the main conclusions would remain the same: timing of maturation and the final body size are intimately related, as demonstrated by the clear and pronounced population differences as well as the strong genetic correlation between the two traits. Moreover, potential differences due to group *vs.* individual rearing are only relevant in the case of the pond population, and should therefore not affect the results from the coastal population (cf. [64]).

Although the statistical evidence for maternal effects was equivocal – most likely due to statistical power issues – the ontogenic patterns observed are worth noting. Maternal effects for body size were suggested to be large in early life stages, and decrease successively such that only a small proportion of variance in older ages was accounted for by maternal effects in both populations. These findings parallel the general patterns observed in earlier studies of fish (e.g. [38], [39]) and suggest that the influence of maternal effects could be an important component of individual fitness in early life stages of nine-spined sticklebacks – assuming of course that body size at that time is associated with fitness. However, although the maternal effects tended to dissipate throughout the duration of the experiment (two months), this pattern may not necessarily hold true in the wild. Specifically, our experimental fish were not exposed to any environmental stress that could amplify and prolong the influence of maternal effects [39]. Further studies are required to verify the significance and to understand the proximate cause of the maternal effects observed here. For example, egg size is an obvious candidate for proximate causes, but other possibilities also exist [39].

Finally, we observed that none of the individuals from the giant-sized population matured in our experiment, raising the obvious question of whether the experimental rearing conditions prevented maturation of fish from this population. Two lines of evidence argue against this possibility. First, Herczeg *et al.* [32] reported that in a similar rearing temperature (17°C) as used here, sexual gonadal differentiation in this population occurred 250 days after hatching. As our experiment only ran until 140 days after hatching, we simply had no possibility of observation of maturation in this population. Second, in our continued rearing of surplus individuals from Pyöreälampi crosses, we observed that some males matured 300 days after hatching (Y. Shimada and T. Shikano, personal observation). Therefore, maturation in the giant pond populations seems to occur naturally much later than in the small-sized coastal population. The ultimate explanation for this late maturation at a larger size has been suggested to be a lack of predation and increased intraspecific competition in the pond as compared to coastal populations [31], [32]. Just like in the case of guppies *Poecilia reticulata* [66], predation is expected to select for earlier maturation at smaller size in coastal populations where nine-spine sticklebacks are faced with predation. However, similar changes could occur also due to other factors. For example, the significant shift towards earlier maturation at a smaller size observed in the North Sea plaice *Pleuronectes platessa* was attributed to changes in temperature [67]. Hence, delayed timing of maturation at a large size in nine-spine sticklebacks might also be influenced by other factors such as local differences in temperature and degree of competition. Therefore, manipulative experiments with temperature- and predation treatments using fish from different populations would facilitate our understanding of the ultimate determinants of gigantism in the nine-spined stickleback. However, regardless of these determinants, timing of maturation appears to provide a key for understanding this. In future experiments, timing of maturation in populations where individuals reach giant-sizes should be clarified by extending the experimental rearing period beyond 300 days.

In conclusion, our results suggest that delayed timing of maturation leads to increased final body size in nine-spined sticklebacks, and provide a proximate explanation for the evolution of gigantism in pond populations of this species. Our results also show that both body size and timing of maturation are moderately to highly heritable traits in this species, and that body size divergence (i.e. *Q*_ST_>*F*_ST_) among populations likely reflects adaptive differentiation caused by natural selection. In general, our results provide illuminating example how two life-history traits - important for individual fitness in ectothermic animals – interact to produce about significant and apparently adaptive differentiation among local populations.

## Materials and Methods

### Sampling and breeding

Adult nine-spined sticklebacks were collected during the early phase of the reproductive period (late May-mid June) in 2008 from two populations: marine fish (small-sized population) from the Baltic Sea in Helsinki (60°13′N; 25°11′E) and freshwater fish from a small pond (Pyöreälampi: large-sized population) in the northeast Finland (66°15′N; 29°26′E). The fish were captured using minnow traps and seine nets with a 6 mm mesh size. Adult fish from the Baltic Sea were transported to the aquaculture facilities of the University of Helsinki, kept under a 24∶0 h (light∶dark) photoperiod and fed with frozen bloodworms (Chironomidae *sp.*). Crosses were made once enough fish from this population had reached reproductive condition. Adult fish from Pyöreälampi were transported to the Oulanka Biological Station (University of Oulu). Crosses were performed within two days and the fertilized clutches were immediately transported to the University of Helsinki.

During 16 June–7 July 2008, 36 sets of paternal half-sib (two females×one male) crosses per population were made artificially by fertilizing the eggs of two different females (72 in total in each population) with sperm from one single male (36 in total). Hence, each male was used to fertilize two independent females, and each of the three parental individuals in the given two crosses was used only once. However, due to mortality and poor fertilization success in some crosses, 28 (Helsinki) and 32 (Pyöreälampi) paternal half-sib crosses were utilized. Eggs were gently squeezed from the ripe females, and a sperm solution was obtained from mincing the testicles of over-anaesthetized males in the ringer solution (NaCl, 170.0 mM; KCl, 6.0 mM; CaCl_2_, 1.6 mM, MgCl_2_, 1.0 mM; pH 6.0). Artificial fertilizations were then performed by adding the sperm solution to the extracted eggs in petri dishes. Eggs were checked regularly and dead or unfertilized eggs were removed. At the eyed-egg stage, eggs were relocated family-wise into 3 L mesh walled tanks within a water bath (12°C: temperature in the wild during the breeding time) with a closed water circulation system with filtering. All offspring hatched eight days after fertilization. Eggs and fish from both populations were reared in fresh water: Pyöreälampi fish are native to freshwater, and the Helsinki fish from the Baltic Sea experience low salinity (ca. 6.0 psu) and often migrate to breed in freshwater. Twenty DAH, an average of 23.2 (range = 10–25) fish from each family were relocated into plastic cages (23×18×27 cm) in duplicate. However, due to logistic constraints, 22 of the Helsinki families were raised in one tank only. Due to the high early mortality among Pyöreälampi fish, the fish density was adjusted down to maximum of 15 fish per tank at 50 DAH. At the end of the experimental period (140 DAH), a total of 544 (10.7 fish / family / tank) fish from Helsinki and 244 (4.2 fish / family / tank) fish from Pyöreälampi were available for analyses.

Fish were fed first with live brine shrimp (*Artemia* sp.) nauplii (2–80 DAHs), then with frozen copepods (*Cyclops* sp.: 50–110 DAHs) and bloodworms (110–140 DAHs). At all stages, food was provided *ad libitum*. Photoperiod was set to twenty-four hour light (natural photoperiod in northern Finland during summer) and water temperature to 17°C throughout the experiment. The experiments were conducted under the licence of the Finnish National Animal Experiment Board.

### Measurements

To monitor growth, the total length of all individuals in each family was measured from photographs taken every 30 days (20, 50, 80, 110 and 140 DAHs), except for two individuals at 110 DAH in Helsinki due to low quality of photographs. All the fish in the given tank were photographed with a digital camera and the total length of each fish was measured – from the tip of nose to the end of fin – to the closest 0.01 mm using the program ImageJ (http://rsbweb.nih.gov/ij/). Maturation and sex of the individuals were also determined from photographs. The sex was identified by the basis of the presence of ventral nuptial coloration in males, and by the presence of ventral abdominal swelling or presence of eggs in ovarian cavity in females. Visual inspection of the data revealed to be conformed to normal distribution (Figure S1 and S2).

### Statistical analyses

To compare growth patterns between the two populations, the measured lengths of the fish at different ages were analysed using linear mixed models (LMEs). In these models, we used total length as the response variable (separate analyses for length at each age, i.e. 20, 50, 80, 110 and 140 DAH). To test the differences in length between the populations, population was considered as a fixed effect (two level factor), and to account for possible variations arising from differences in fish density in rearing tanks, density at the focal and previous measurement time-points were accounted for as fixed covariates. Block (i.e. replicate) nested in family was considered as a random effect. To compare total lengths of females, males and immature fish at 80, 110 and 140 DAHs in the Helsinki population, LMEs were fitted with total length as the response variable (again, separate analyses for each age), sex as a category (female, male or immature) and density as fixed effects, and block nested in family as random effects. The determinants of the onset of maturation were investigated using generalized linear models (GLM) with a binomial error structure. The frequencies of mature and immature individuals in each family at 80 DAH were modelled by having length and density at 50 and 80 DAH as well as population as fixed effects. A similar analysis was also performed for the numbers of mature and immature individuals at 110 DAH, so that length at and density at 50, 80 and 110 DAH as well as population were considered as additional covariates. All of the above models were reduced stepwise-fashion by comparing likelihood ratio test (LRT) in case of LMEs and deviance (D) in the case of GLM. Analyses were preformed with R 2.10.1 [68].

### Estimation of genetic parameters, genetic correlation and quantitative genetic differentiation

An animal model approach utilizing GLMMs with Markov chain Monte Carlo (MCMC) techniques was used for genetic parameter estimation [69]. Here, an additive genetic variance component term is fitted directly considering the relatedness between individuals using a numerator relationship matrix based on a pedigree file [70]. In our analyses, we accounted for variance due to fish density by adding it as covariate in the models, as well for sex effects by adding sex as a fixed effect. The sex term had three levels (*viz.* male, female and immature) as we could not distinguish the sex of immature fish. A block term was fitted to account for variance among replicate tanks. Additive genetic (σ^2^_a_), maternal (σ^2^_m_) and residual (σ^2^_e_) variances for body size in both populations and timing of maturation in Helsinki population, were estimated using the following mixed linear model:
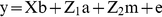
(1)where y is the vector of phenotypic observations, b is the vector of fixed effects (overall mean, density, block and sex), a is the vector of random additive genetic effects, m is the vector of random maternal effects (both genetic and environmental effects, see [71]) and e is the vector of random residuals (environmental and non-additive effects). X, Z_1_ and Z_2_ are design matrices linking the phenotypic observations with the fixed and random effects. Random effects were assumed to follow a multivariate normal distribution (see [70]). Narrow-sense heritability (*h* ^2^) and maternal effects (*m* ^2^) estimates were calculated for body size and timing of maturation (2 and 3, respectively) as follows:
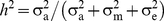
(2)

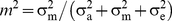
(3)The estimations were performed using the R package MCMCglmm [69] in R 2.10.1 (http://cran.r-project.org/). We opted to use MCMCglmm, because Bayesian methods have generally proven more conservative and are less likely to underestimate standard errors than non-Bayesian methods [e.g. 72]. The significance of random factors was tested using a deviance information criterion (DIC) comparing the full model with its appropriate restricted model (i.e. dropping the effect and comparing the fits of the two models, see [37]).

In estimation of heritability and genetic correlations, we assumed normal distribution and used uniform priors following Alho *et al.* [73]. The posterior distributions of the model parameters were estimated via MCMC runs, with a chain length of 2×10^5^ iterations for the two traits (size and timing of maturation) and genetic correlation between them. Of these, 1000 were sampled. The parameter estimates quoted are the mean of these 1000 samples, and the 95% credible interval (C.I.) is the region with the 95% lowest and highest posterior density.

The estimates of the indices of quantitative genetic differentiation (*Q*_ST_; [74]) were estimated by Bayesian inference using OpenBUGS version 3.1.2 [75]. Modelling of *Q*_ST_ was done as in Cano *et al.* [76]. For all the analyses the posterior distributions were obtained by running two chains of 10,000 iterations each. After a burn-in of 5000 iterations, when convergence was reached, every second iteration was taken to give 2×5000 draws from the posterior distribution. The degree of neutral differentiation was quantified using the standardized variance in allele frequencies (*F*_ST_) as estimated by θ [77]. Standard errors of *F*_ST_ were obtained by jackknifing over loci and significance tests were performed by 1000 permutations. The *F*_ST_ estimation and its significance testing were done using FSTAT 2.9.3 [78]. The genotype data for *F*_ST_-estimation was produced by Shikano *et al.* [62], from which data on allele frequency variation in 104 microsatellite loci was available. To test neutrality of these marker loci, outlier analyses were conducted using LOSITAN [79], which is widely employed for the detection of loci under directional and balancing selection [80]. Simulation parameters were set according to Shikano *et al.* [81]. Since one locus (Ppgm35) was indicated to be under balancing selection (*P*<0.05), this locus was excluded from the estimation of neutral population differentiation. Accordingly, *F*_ST_ was calculated using 103 putatively neutral loci with 24 fish from Helsinki and 24 fish from Pyöreälampi population that were parts of the parental fish used for crosses in this study. When densities at the focal and previous measurement time-points were initially accounted for as covariates at the heritability estimation, a few of estimates had convergence problems. Therefore, we used the mean density during previous-to-focal period as a covariate in both of heritability and *Q*_ST_ estimation, except at 20 DAH, when they could be always fitted separately.

## Supporting Information

Figure S1 **Mean total length (mm) of the experimental fish by family at five different time points in two populations.** Bars show standard deviation. Bar graphs show the frequency distribution of body size within each population at given time point.
(TIF)

Figure S2 **Percentage of matured fish by family at three different time points in Helsinki population.** Bar graphs show the frequency distribution of percentage of matured individuals at given time point across different families.
(TIF)
