# Supplementary figures and images for: Quantitative Genetics of Body Size and Timing of Maturation in Two Nine-Spined Stickleback (Pungitius pungitius) Populations
Source: PLoS One. 2011 Dec 14;6(12):e28859. doi: 10.1371/journal.pone.0028859 (PMC3237540; doi:10.1371/journal.pone.0028859)

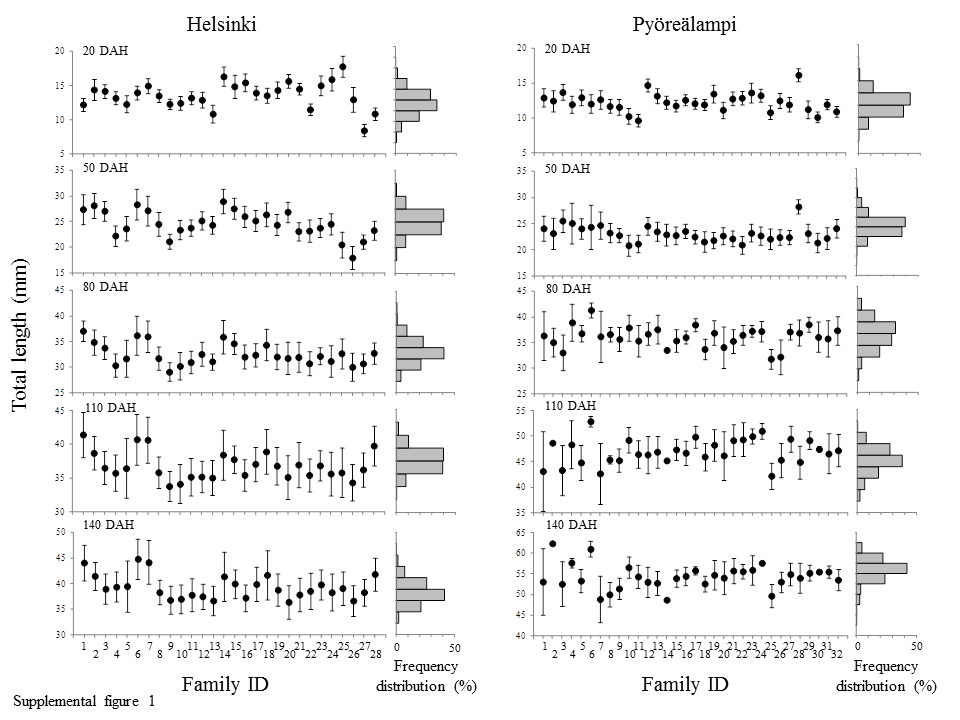

Supplement: Figure S1 — Mean total length (mm) of the experimental fish by family at five different time points in two populations. Bars show standard deviation. Bar graphs show the frequency distribution of body size within each population at given time point. (TIF) [file pone.0028859.s001.tif]

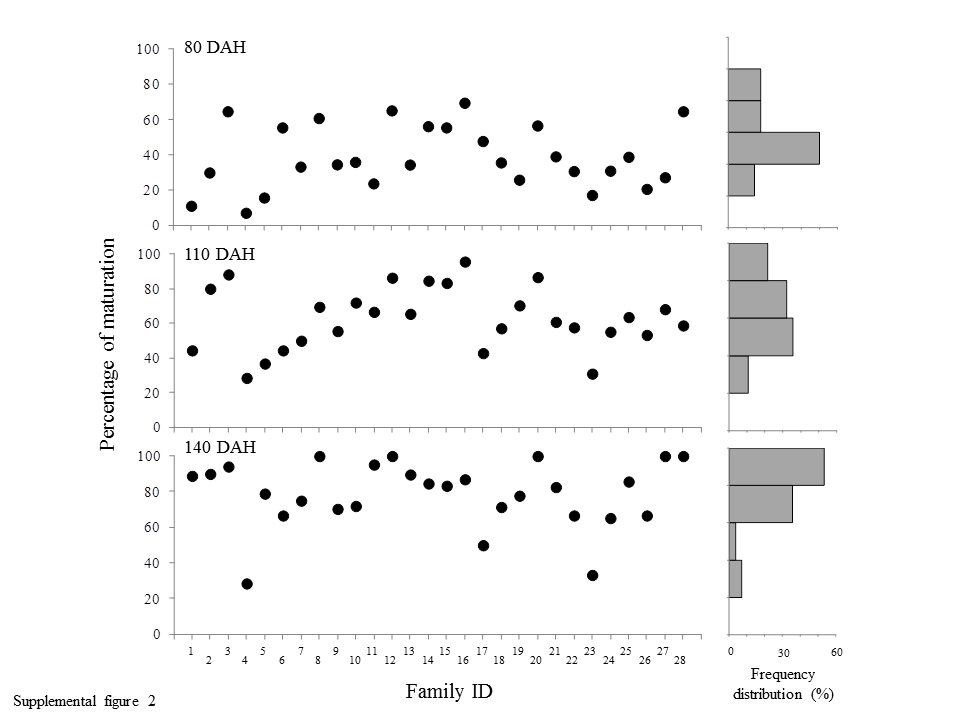

Supplement: Figure S2 — Percentage of matured fish by family at three different time points in Helsinki population. Bar graphs show the frequency distribution of percentage of matured individuals at given time point across different families. (TIF) [file pone.0028859.s002.tif]
